# Subgenotypes and Mutations in the S and Polymerase Genes of Hepatitis B Virus Carriers in the West Bank, Palestine

**DOI:** 10.1371/journal.pone.0113821

**Published:** 2014-12-12

**Authors:** Zakeih Abdelnabi, Niveen Saleh, Sabri Baraghithi, Dieter Glebe, Maysa Azzeh

**Affiliations:** 1 Virology Research Laboratory, Medical Research Center, Al-Quds University, Abu Dies-East Jerusalem, Palestine; 2 Al-Makassed Islamic Charitable Hospital (MICH) Central Laboratory, East Jerusalem, Palestine; 3 Institute of Medical Virology, Justus-Liebig University Giessen, National Reference Center for Hepatitis B and D Viruses, German Center for Infection Research (DZIF), Biomedical Research Center, Giessen, Germany; Yonsei University College of Medicine, Republic of Korea

## Abstract

The mutation rate and genetic variability of hepatitis B virus (HBV) are crucial factors for efficient treatment and successful vaccination against HBV. Until today, genetic properties of this virus among the Palestinian population remain unknown. Therefore, we performed genetic analysis of the overlapping S and polymerase genes of HBV, isolated from 40 Palestinian patients' sera. All patients were HBsAg positive and presented with a viral load above 10^5^ HBV genome copies/ml. The genotyping results of the S gene demonstrated that HBV D1 was detected in 90% of the samples representing the most prominent subgenotype among Palestinians carrying HBV. Various mutations existed within the S gene; in five patients four known escape mutations including the common G145R and D144E were found. Furthermore, a ratio of 4.25 of non-synonymous to synonymous mutations in the S gene indicated a strong selection pressure on the HBs antigen loops of HBV strains circulating in those Palestinian patients. Although all patients were treatment-naïve, with the exception of one, several mutations were found in the HBV polymerase gene, but none pointed to drug resistance. The study presented here is the first report to address subgenotypes and mutation analyses of HBV S and polymerase genes in Palestine.

## Introduction

HBV infection remains a health problem worldwide with over two billion infected people and 600 000 deaths yearly [1]. Efficient treatment and vaccination strategies are persisting challenges due to genetic heterogeneity of HBV DNA. The HBV DNA is only 3.2 kb long with four open reading frames encoding seven viral proteins, two of which are the viral polymerase and the small HBV surface (S) protein which is also named hepatitis B surface antigen (HBsAg). According to the overall nucleotide sequence variations of the entire genome, HBV is classified into nine genotypes (A-I) differing by at least 8% of the DNA sequence [2], [3]. These genotypes are furthermore divided into different subgenotypes that differ by at least 4% and are referred to with numbers [4]. Subgenotype distribution varies with geographic location; while subgenotype A2 is more common in northern Europe and the USA, A1 and other A subgenotypes are more prevalent in Africa. Genotypes B and C are prevalent in East and Southeast Asia, while D is described to be predominant in the Mediterranean, Near East and Oceania, beside its worldwide distribution [2], [3].

Many studies on HBV subgenotypes analyze only the S gene, which is usually sufficient for accurate typing. At the antigen level, HBsAg is divided into nine major subtypes according to the combination of its common antigen determinant *a* with the subtype determinants *d* or *y, w1-4* or *r*: *adw2, adw3, adw4, adr, ayw1, ayw2, ayw3, ayw4*, and *ayr* [5]. The common antigenic *a* determinant in the S gene product is conserved in “normal” HBV strains and formed by conformational epitopes of the amino acids 124–147 [6], [7], [8]. Further heterogeneity is caused by point mutations, deletions and by genetic recombination with pre-S genes of different HBV strains [9]. HBV-infected recipients of hepatitis B vaccines or occult infected HBV carriers, who develop protective anti-HBs antibodies, may evoke HBV mutants encoding HBsAg with a more or less altered *a* determinant or untypical subtype determinants [10], [11], [12]. Such mutants can escape the host immune responses, and are therefore called “escape mutants”.

While the N-terminal domain of the viral polymerase forms the terminal protein (TP) linked to the viral DNA, its central domain forms the reverse transcriptase (RT), the coding region of which is largely overlapped by the S gene. The viral RT is an error prone-enzyme, as it lacks a proof reading function, producing HBV mixture of mutants and wild type. Therefore, mutations occur quite often and may be selected for during antiviral therapy [13]. The mutation rate of HBV is 10 times higher than that known for other DNA viruses, and is almost as high as that known for the retrovirus HIV [14], [15]. The HIV- and HBV-RT inhibiting drug, Lamivudine, is still widely used and is the only drug made available by the Palestinian Ministry of Health for antiviral treatment of HBV-infected patients. However, the highest resistance among licensed HBV antivirals has been attributed to Lamivudine with a yearly rate of 14-32%, reaching 70% after four years of treatment [16]. Primary mutations causing Lamivudine resistance are located within the tyrosine-methionine-aspartate-aspartate (YMDD) motif of the viral pol/RT reading frame. An acute hepatitis B infection does not necessarily require therapy, as 90-95% of adults resolve the acute HBV infection and develop immunity [17]. However, children are at much higher risk for chronic infection after exposure to HBV [18].

Using tools of molecular biology and bioinformatics, we analyzed the S and RT gene regions of HBV from 40 Palestinian HBV-patients. To the best of our knowledge, this study is the first analysis of mutations and variations within the S and RT genes of HBV conducted in Palestine.

## Methods

### Ethics statement

Patients' data collection, using archived patients' samples and performing this research were approved by Al-Makassed Islamic Charitable Hospital (MICH) and Al-Quds University ethics committees.

### Patients' samples

200 HBsAg positive serum samples from MICH were archived and subjected to viral load testing at the Virology Research Laboratory (VRL), Medical Research Center (MRC), Al-Quds University, Abu Dies, East Jerusalem, Palestine, where this research took place. HBsAg test was performed at MICH Central Laboratory using AxSYM kit HBsAg V2 on the Abbott AxSYM machine according to manufacturer instructions. MICH is the referral hospital for Palestine and the main teaching hospital associated with Al-Quds University Medical School. The archived sera were remnants of tested patients' samples, which otherwise would have been discarded. Serum samples presented with a viral load above 10^5^ HBV genome copies/ml were subjected to genotyping. In all cases, patient's names' were substituted by “AQ-number” codes (AQ-1 to AQ-40). Age, sex and residency of patients were mainly retrieved from patient's medical file based on MICH approval for archived samples. Once a child was tested positive for HBsAg, parents or guardians were questioned personally regarding the vaccination circumstances. Requests for parents' serum samples were made orally.

### HBV DNA extraction and viral load

HBV DNA was extracted from 200 µl serum using QIAamp DNA Mini kit (51304, Qiagen, Germany) according to manufacturer's instructions. Real-time PCR (rt-PCR) was performed using an ABI Real Time PCR 7500 system (Applied Biosystems, USA). 2.5% of the 50 µl extracted DNA and TaqMan universal master mix (4304437, Applied Biosystems, USA) were used for amplification. The amplification targeted the x gene of the HBV using specific primer pair (XF: 5'-GAC GTC CTT TGT YTA CGT CCC GTC- 3', XR: 5'- TGC AGA GGT GAA GCG AAG TGCACA- 3') and probe (FAM 5'- ACG GGG CGC ACC TCT CTT TAC GCG G-3' –MGB [19]. Validated complete genome HBV-DNA (REF. 05960116, Clonit, Italy) at 10^6^, 10^5^, 10^4^, 10^3^, 10^2^ and 10^1^ copies/µl was utilized as standard in all rt-PCR assays. All standards, negative controls, and test samples were tested in duplicate manner. The amplification reaction started with 2 min at 50°C, followed by 10 min at 95°C and final 45 cycles as following: 95°C for 15 sec and 60°C for 1 min.

### Amplification of the S and polymerase genes

Primer pair S6 sense (5′-TGG ATG TGT CTG CGG C-3′) and S6 antisense (5′-CKT TGA ACA DAC TTT CCA ATC AAT AG-3′) (validated primer sequences from the Institute of Medical Virology, National Reference Center for Hepatitis B and D Viruses, German Center for Infection Research (DZIF), University of Giessen, Germany) were used to amplify a 621 bp DNA fragment spanning the S and polymerase gene regions. Advantage 2 polymerase mix (639201, Clonetech, USA) was used for the amplification, which started with a single hot start step for 3 min at 95 °C followed by 40 cycles in the following order, 30 sec at 95 °C, 45 sec at 58 °C, and 1 min at 68 °C respectively. An additional extension step was performed for another 5 min at 68 °C. The PCR product was purified using Mini Elute PCR purification Kit (28004, Qiagen, Germany) according to manufacturer's instructions. Sequencing was performed using Sanger chemistry (ABI PRISM 3130 Genetic Analyzer; Applied Biosystems, USA).

### HBV Sequence analysis and typing

Sequences obtained with the forward and reverse primer were used for the sequence analysis of each PCR product belonging to each patient. Sequences were subjected to the NCBI BLAST search for initial identification of the HBV genotype (http://www.ncbi.nlm.nih.gov/blast/Blast.cgi?PAGE=Nucleotides). For accurate sequence analysis, forward and reverse sequences were aligned with NCBI archived complete HBV genome sequences, representing the genotype identified in NCBI blast search, using the DNASTAR Lasergene MegAlign program (DNASTAR Inc., WI, USA). Lasergene MegAlign program marks nucleotides' differences between the sequence set as reference and our sequences in red. Thereafter, the sequencing chromatograms of our sequences were inspected visually for these nucleotides and corrected if necessary. This procedure is essential to verify whether a detected nucleotide difference was due to real mutation or to a technical problem in the automatic chromatogram readings.

The corrected Palestinian sequences of the same subgenotype were entered in a new DNASTAR Lasergene MegAlign file, along with different NCBI archived sequences of the same subgenotype. S and polymerase genes for each Palestinian sequence were analyzed separately; synonymous and non-synonymous mutations for each sequence were recorded and searched for through scientific literature. The annotated 40 sequences (AQ-1 to AQ-40) were submitted to GenBank and assigned the accession numbers KC528610-KC528649.

## Results

### General patients' data and demographics

Palestinian patients subjected to HBV subgenotype analysis were from all over the West Bank with 27.5% from southern Palestine (Hebron and Bethlehem districts), 45% from the middle (East Jerusalem and Ramallah districts) and 27.5% from northern Palestine (Nablus, Jenin, Tulkarem and Qalqilya districts). The patients were between 8 months and 80 years old, distributed between 57.5% males to 42.5% females. Two samples from children were tested along with those of their mothers.

### HBV infected children of the vaccination era

Of the 40 samples, five (12.5%) belonged to children at the age of 8 months, three years (two children), five and nine years. None of these children was delivered at MICH, which follows a strict TORCH test policy for pregnant women. All five children were infected with HBV subgenotype D1. With the exception of the eight-month-old child, the other four children were tested for HBsAg, as they were scheduled for surgery. Mothers of the eight-month and three-year-old children also had subgenotype D1 and both had sequences 99% identical with their children. The mother of the eight-month-old baby was not aware of her HBV infection; the child received the first two doses of hepatitis B vaccine (Engerix, GSK) but not HBIG and missed the third vaccine dose. The second mother was aware of her HBV infection and assured us the child did indeed receive the three doses of vaccine. Mother of the five-year-old child tested positive for HBsAg and presented with HBV viral load below 100 genome copies/ml. Guardians of the five, nine, and the other three-year-old children assured us that they received all three doses of active vaccination.

### HBV genotypes and subgenotypes in the Palestinian patients

The analysis of the S gene from the 40 Palestinian samples revealed that 36 (90%) represented the D1 subgenotype. One (2.5%) sample belonged to the D3 subgenotype and three (7.5%) to the A2 subgenotype respectively.

### Mutation analysis of the S gene in Palestinian HBV isolates

Analysis of the S gene in the 36 Palestinian D1 genotype isolates revealed 19 non-synonymous mutations, resulting in 17 different amino acid exchanges, six of which were located in the *a* determinant (Table 1). Most of these mutations (14) occurred in single cases, three occurred twice and two occurred three times, respectively. Simultaneously, seven synonymous mutations were also detected in the same 36 Palestinian D1 isolates (Table 2).

**Table 1:** Non-synonymous mutations in the S region of Palestinian D1 subgenotypes.

nt position	aa position	Occurrence	Reported function/detected in	Reference
410:A/T	I86F	1	Unknown/Chronic HBV carriers with D1 subgenotype	[63]
429:T/C	I92T	1	Unknown/Subgenotype C1	[64]
482:A/C	I110L	1	Unknown/solely anti-HBc-positive sera, genotype A	[34]
484:T/G	I110L	1	Unknown/solely anti-HBc-positive sera, genotype A	[34]
**531: C/G**	**T126S**	**1**	**Escape mutation**	[35]
**533: C/T**	**P127S**	**1**	**Escape mutation**	[36]
555:A/T	Y134F	2	Unknown/solely anti-HBc- positive sera, genotype D	[34]
581:T/A	S143T	1	Unknown/solely anti-HBc-positive sera, genotype D	[34]
**586: C/A**	**D144E**	**2**	**Escape mutation**	[37]
**587: G/A**	**G145R**	**3**	**Escape mutation**	[38]
720:C/T	T189I	1	Reduces HBsAg detection signal of genotype E	[65]
753:A/C	Y200F	1	Unknown/naïve patients	[64]
765:G/A	S204R	1	Unknown/genotype E	[34]
771:A/T	Y206L	1	Unknown	Novel
772:G/T	Y206L	1	Unknown	Novel
774:G/A	S207N	2	Unknown/solely anti-HBc-positive sera, genotype D	[34]
784:T/A	S210R	1	Unknown/genotype A	[34]
791:T/A	L213I	3	Unknown/genotypes D and C	[34]
791:T/A	L213F	1	Unknown/solely anti-HBc-positive sera, genotype D	[34]

Positions of the nucleotide (nt) mutation and the correlating amino acid (aa) are presented based on their location in the archived GenBank reference DQ315778. Occurrence reflects the number of samples (patients) in which the mutation was detected. Known escape mutations are in boldface. Exchanges marked with (*) are considered polymorphisms due to their prevalence in>10% of the 40 patients. Corresponding references and proposed functions are provided in the last two columns. Unreported mutations were considered novel.

**Table 2:** Synonymous mutations in the S region of Palestinian D1 subgenotypes.

nt position	aa position	Occurrence
457:A/G	Q101Q	2
493:T/(A,C,G)	S113S	11*
499:T/(C,A)	T115T	7*
538:T/A	A123A	2
562:C/A	S136S	1
619:C/T	S155S	4*
784:T/C	S210S	1

Positions of the nucleotide (nt) mutation and the correlating amino acid (aa) are presented based on their location in the archived GenBank reference DQ315778. Occurrence reflects the number of samples (patients) in which the mutation was detected. Exchanges marked with (*) are considered polymorphisms due to their prevalence in>10% of the 40 patients.

The only Palestinian D3 case showed six non-synonymous mutations, one of which was in the *a* determinant, and three synonymous mutations (Tables 3 and 4).

**Table 3:** Non-synonymous mutations in the S region of Palestinian D3 subgenotype.

nt position	aa position	Reported function/detected in	Reference
528: C/T	T125M	Increases HBsAg reactivity in immunological diagnostic assays	[66]
753: A/T	Y200F	Unknown/antiviral therapy	[64]
762: C/A	P203Q	Causes discrepant results in some HBsAg detection assays	[67]
766: T/A	S204R	Unknown, genotype E	[34]
770:T/A, 771: A/C	Y206T	Unknown	Novel
774: G/A	S207N	Unknown/solely anti-HBc-positive sera, genotype D	[34]

Positions of the nucleotide (nt) mutation and the correlating amino acid (aa) are presented based on their location in the archived GenBank reference JF754625. Corresponding references and proposed functions are provided in the last two columns. Unreported mutations were considered novel.

**Table 4:** Synonymous mutations in the S region of Palestinian D3 subgenotype.

nt position	aa position
532:T/C	T126T
562:C/A	S136S
616:A/G	S154S

Positions of the nucleotide (nt) mutation and the correlating amino acid (aa) are presented based on their location in the archived GenBank reference JF754625.

L209V was the only amino acid exchange detected in the S gene of the three A2 Palestinian genotypes, due to T779G point mutation. Two synonymous mutations were found in one of the three samples at position C406T (L84) and A436G (L94). The nucleotide position was defined based on A2 subgenotype GenBank archived sequence X51970.

### Mutations occurring in the RT gene in Palestinian HBV isolates

Twenty-three non-synonymous RT gene mutations were recorded in the 36 D1 Palestinian HBV isolates (Table 5). Most mutations were caused by single nucleotide substitutions. However, the F122I mutation was caused by three different nucleotide substitutions at position 493. Three further mutations; H124Y, S219P and I266K were caused by two different nucleotide substitutions at positions 499, 784 and 926 respectively (Table 5). Eleven synonymous gene mutations were recorded in the RT gene region of the 36 Palestinian D1 samples (Table 6). C619T (L169) was the most common synonymous mutation, occurring in four samples (Table 6). Other synonymous mutations occurred in 3, 2, or 1 sequence respectively.

**Table 5:** Non-synonymous mutations in the RT region of Palestinian D1 subgenotypes.

nt position	aa position	Occurrence	Reported function/detected in	Reference
400:C/A	L91I	1	Unknown/patients treated with NRTIs	[68]
410:A/T	H94I	1	Unknown	Novel
457:A/G	R110G	2	Unknown/naïve patients	[69]
472:T/G	L115V	1	Unknown/naïve patients	[70]
493:T/(A,C,G)	F122I	15*	Unknown	[71]
499:T/(C,A)	H124Y	9*	Unknown	[71]
533:A/C	Y135S	35*	Unknown/HIV-positive HBV patient after LVD therapy	[72]
533:A/T	Y135F	1	Unknown	Novel
538:T/A	S137T	2	Unknown	[71]
562:C/A	L145M	1	Unknown/naïve patient	[70]
586:C/A	R153K	2	Proposed to enhance viral polymerase fitness	[72]
587:G/A	R153Q	2	Reduces the replication efficiency of the viral polymerase.	[72]
784:T/(C,A)	S219P	2	Unknown/ETV-treated patients	[69]
791:T/A	F221Y	3	Unknown/naïve patients	[73]
793:A/T	T222S	1	Unknown/ETV-treated patients	[69]
823:C/A	P237T	1	Unknown/naïve patients	[73]
871:A/C	N248H	24*	Unknown/HIV-positive HBV patient after LVD therapy	[72]
895:T/A	C256S	2	Unknown	[71]
918:T/A	D263E	1	Unknown	[71]
926:T/(G,A)	I266K	3	Unknown/patients treated with NRTIs	[68]
950:G/A	R274K	4*	Unknown/NRTIs–naïve patients	[68]
965:A/C	N279T	1	Unknown	GenBank AB106564
1055:T/A	M309K	2	Impairs viral polymerase activity	[74]

Positions of the nucleotide (nt) mutation and the correlating amino acid (aa) are presented based on their location in the archived GenBank reference DQ315778. Occurrence reflects the number of samples (patients) in which the mutation was detected. Corresponding references and proposed functions are provided in the last two columns. Unreported mutations were considered novel. Exchanges marked with (*) are considered polymorphisms due to their prevalence in>10% of the 40 patients. NRTIs: nucleoside and/or nucleotide reverse-transcriptase inhibitors, LVD: Lamivudine, ETV:Entecavir.

**Table 6:** Synonymous mutations in the RT region of Palestinian D1 subgenotypes.

nt position	aa position	Occurrence
555:A/T	V142V	2
619:C/T	L168L	4*
720:C/T	H117H	1
774:G/A	Q215Q	2
853:A/C	R242R	1
888:C/A	V253V	2
906:A/C	S259S	1
907:T/(A,C)	L260L	2
909:G/A	L260L	1
969:G/A	R280R	1
987:C/(G,T,A)	V286V	3

Positions of the nucleotide (nt) mutation and the correlating amino acid (aa) are presented based on their location in the archived GenBank reference DQ315778. Occurrence reflects the number of samples (patients) in which the mutation was detected. Exchanges marked with (*) are considered polymorphisms due to their prevalence in>10% of the 40 patients.

Seven non-synonymous mutations and six synonymous mutations were found in the RT region of the only Palestinian D3 isolate (Tables 7 and 8).

**Table 7:** Non-synonymous mutations in the RT region of Palestinian D3 subgenotypes.

nt position	aa position	Reported Function/detected in	Reference
532:T/C	Y135H	Unknown/naïve patients	[69]
562:C/A	L145M	Unknown/naïve patients	[70]
616:A/G	I163V	Unknown	[71]
766:T/A	S213T	Candidate mutation associated with hepatocellular carcinoma	[74]
770:T/A	V214D	Unknown/naïve patient	GenBank:FJ904404
895:T/A	C256S	Unknown	[71]
926:T/A	I266K	Unknown/patients treated with NRTIs	[68]

Positions of the nucleotide (nt) mutation and the correlating amino acid (aa) are presented based on their location in the archived GenBank reference JF754625. Corresponding references and proposed functions are provided in the last two columns. NRTIs: nucleoside and/or nucleotide reverse-transcriptase inhibitors.

**Table 8:** Synonymous mutations in the RT region of Palestinian D3 subgenotypes.

nt position	aa position
528:C/T	H134H
753:A/T	V208V
762:C/A	A211A
774:G/A	Q215Q
852:G/A	K241K
969:G/A	R280R

Positions of the nucleotide (nt) mutation and the correlating amino acid (aa) are presented based on their location in the archived GenBank reference JF754625.

Six non-synonymous mutations and five synonymous mutations were detected in the RT gene region of the three Palestinian A2 samples (Tables 9 and 10). Non-synonymous mutations L217R and I253V and synonymous mutations Y252, G258, and K268 were present in all A2 samples.

**Table 9:** Non-synonymous mutations in the RT region of Palestinian A2 subgenotypes.

nt position	aa position	Occurrence	Reported function/detected in	Reference
406:C/T	L93F	1	Unknown	Novel
436:A/G	I103V	1	Unknown	GenBank AF143307
779:G/T	L217R	3	Unknown	[71]
886:A/G	I253V	3	Unknown	[71]
950:G/A	R274K	1	Unknown/NRTIs–naïve patients	[68]
952:A/G	K275E	1	Unknown/ETV-treated patients	[69]

Positions of the nucleotide (nt) mutation and the correlating amino acid (aa) are presented based on their location in the archived GenBank reference X51970. Corresponding references and proposed functions are provided in the last two columns. Unreported mutations were considered novel. NRTIs: nucleoside and/or nucleotide reverse-transcriptase inhibitors, ETV: Entecavir.

**Table 10:** Synonymous mutations in the RT region of Palestinian A2 subgenotypes.

nt position	aa position	Occurrence
885:C/T	Y252Y	3
903:A/G	G258G	3
933:G/A	K268K	3
987:A/C	V286V	2
994:A/C	R289R	2

Positions of the nucleotide (nt) mutation and the correlating amino acid (aa) are presented based on their location in the archived GenBank reference X51970.

### Polymorphism and the ratio of non-synonymous to synonymous mutations

Seventeen non-synonymous and seven synonymous mutations were found in the S gene of the 36 Palestinian D1 subgenotypes (Tables 1 and 2). Three of the synonymous mutations were referred to as polymorphism due to their occurrence in more than 10% of the total 40 samples (Table 2). The ratio of non-synonymous (17) to synonymous non polymorphic mutation (4) in the S gene of the D1 subgenotypes was 4.25.

Eleven synonymous mutations were found in the RT region of D1 subgenotypes (Table 6). One of these synonymous mutations and five of the 23 non-synonymous mutations were classified as polymorphisms, as they were prevalent in more than 10% of the 40 total samples (Tables 5 and 6). The ratio of non-synonymous (18) to synonymous (10) mutations without polymorphism was 1.8, which is significantly less than that for the S region.

## Discussion

HBV genotype D was the most prominent among Palestinian patients. Only 7.5% of the samples had genotype A. The predominance of genotype D is consistent with regional reports from Egypt, Jordan, Israel, Syria, and Lebanon [20], [21], [22], [23], [24], [25]. Genotype D has a worldwide distribution; but it is predominant in some regions, while a minor component in others. Regions of high genotype D prevalence occur in the Mediterranean and in large parts of Asia, except East and South East Asia [2]. The most prominent subgenotype among Palestinians was D1, detected in 90% of the samples. Subgenotype D1 is the most common subgenotype in Turkey, Greece, Iran, Pakistan, Egypt, Lebanon, Israel and others [2], [25], [26], [27], [28], [29], [30], [31]. One single Palestinian sample belonged to D3 subgenotype. Subgenotype D3 is found prominently in Europe [2], but some regional studies report a low prevalence of D3 subgenotype [26], [27]. Surprisingly three Palestinian samples belonged to subgenotype A2. Subgenotype A2 is common in Northern and Central Europe and in the European offspring of Caucasians living in South Africa and USA [2]. Genotype A is barely reported in the region, with the exception of one report from Egypt, where a mix of genotypes D and A were detected in pediatric cancer patients [21]. Interestingly, one of the three patients with A2 genotype had received blood transfusion. We propose that this blood was not donated from local donor but rather from a foreign donor. However, we were unable to obtain further information regarding this or regarding the other two A2 samples.

The ratio of non-synonymous to synonymous non-polymorphic mutations is an indicator for the evolutionary relevance of a set of mutations [32]. Ratios below 1 imply that these mutations are genetically neutral as suggested by Gojobori et al. (1990) for HBV and other viruses [14]. A ratio of 4.25 for the S genes indicates a strong selection effect on HBV strains circulating in the Palestinian patients that were studied. Evidence for this selection may be the amino acid exchanges I110L and Y206L; each occurred twice in Palestinian patients and was caused each time by different nucleotide substitution events. The selection affected only the two last third of the S gene upstream of aa85, while the transmembrane helix I and the internal loop of HBsAg were completely conserved.

Six of the non-synonymous mutations in the D1 subgenotypes were found in the *a* determinant of the S gene, three were found upstream and eight downstream the *a* determinant respectively. Table 1 shows that the mutations found upstream–I86F, I92T, and I110L–and those found downstream–T189I, Y200F, S204R, Y206L, S207N, S210R, L213I, and L213F–were recorded in different studies but were not associated with critical functions (Table 1).

The six mutations found in the *a* determinant displayed different characteristics. The amino acid exchange Y134F found in two patients correlated either with the HBsAg genotype A/adw2 [33] or genotype D/ayw2 [34]. S143 is typical for genotype A and T143 for genotype D [34]. The other four mutations found in the *a* determinant–T126S, P127S, D144E, and G145R–are known escape mutants [35], [36], [37], [38] and were detected in five Palestinian D1 samples accounting for 12.5% of the total 40 samples. Two of these samples presented with the escape mutations D144E and G145R simultaneously, while the other three samples presented with one single escape mutation each; G145R, T126S, or P127S.

Mutations in the *a* determinant of the S gene occur frequently in occult HBV infection, and are a potential risk to blood safety [39], [40]. This is the case, when HBsAg is seemingly absent in the presence of HBV DNA in serum, which was, however, not the case in our sample. Previous HBsAg assays often failed to detect HBsAg with mutations in the HBsAg loop but last generation assays can detect most HBsAg escape mutants if they are present in sufficient concentration [41], [42]. Different studies demonstrate that HBV viruses carrying antibody escape mutations have reduced binding affinity of anti-HBs antibodies to HBsAg, including vaccine generated antibodies, which may allow infection despite vaccination [10], [12], [35], [36], [43], [44]. All escape mutants detected in our study were found in patients, who were above 36 years of age, which means they were not subjected to vaccination. It is probably that an unrecognized antibody response of the patients against their own HBsAg had exerted some selective pressure in favor of classical escape mutants and mutations to an amino acid associated with another genotype like Y134F and S143T. According to earlier reports, S gene mutations accumulate in chronic HBV infection, particularly after development of immune pathogenesis or loss of HBeAg [45].

None of the six non-synonymous mutations found in the only Palestinian D3 sample was attributed to critical functional impact on viral activity (Table 3), in contrast, L209V, the only mutation found in the S gene of all three Palestinian A2 samples was reported in transplant recipients, who received HBIG [33] and in vaccinated individuals [46]. In the second report, authors propose that antibodies produced due to vaccination may not be effective in neutralizing HBV mutants including the L209V in genotype E [47].

Twenty-three, seven and six non-synonymous mutations were detected in the RT region of the Palestinian D1, D3, and A2 isolates respectively (Tables 5, 7, and 9). Although few of these mutations affect the viral polymerase activity, none was reported to confer drug resistance (Tables 5, 7, and 9).

Out of the 40 samples analyzed here, five belonged to children, who were vaccinated against HBV. These children were born at Palestinian public hospitals. Contrary to MICH policy and that of private clinics in Palestine, screening pregnant women for HBsAg and so administrating HBIG immediately after birth does not belong to the policy of the Palestinian Ministry of Health. Based on our data, we assume that all five children were suffering from breakthrough infections. Indeed, this is proven for the two cases with the children HBV sequences almost identical to their mothers. Breakthrough is strongly suggested in the five-year-old child as the mother tested positive for both HBsAg and HBV DNA. It had been shown earlier that the rate of perinatal transmission of HBV from infected mothers to offspring varies from 5 % to 31 % in HBsAg positive mothers to 85 % to over 90 % in HBeAg positive mothers or those presenting with high viral load [48], [49], [50], [51], [52], [53]. One factor favoring breakthrough in spite of immediate active vaccination at birth may be the fact that the vaccine has subgenotype A2 and HBsAg adw2, whereas the transmitted HBV in these cases were D1/ayw2. Vaccine-induced protection seems to be weaker against heterologous genotypes [18]. The fact that these children did not receive HBIG may have contributed to the breakthrough, even though the additional effect of HBIG to active vaccination has not been fully established [54]. It is widely accepted and recommended that the most efficient precaution for prevention of perinatal HBV transmission is screening pregnant women for HBsAg and the immediate administration of one infant dose of hepatitis B immune globulin (HBIG) along with the first hepatitis B vaccine dose within 12 hours after birth, followed by the second and the third vaccine dose at 1 and 6 month of age [55], [56], [57], [58], [59].

Screening mothers worldwide leads to significant reduction in the infection of their children [53], [60], [61], [62]. We hope that addressing this critical factor can advocate the introduction of screening pregnant women for HBsAg all over Palestine.

Finally, identifying specific genotypes and mutants of pathogens in different geographic areas is crucial for understanding clinical severity of the infection and developing control measures according to the predominant local strains, and can contribute to optimization of therapy, vaccines and diagnostic tools.
